# Surgical and Orthodontic Management of Fused Maxillary Central and Lateral Incisors in Early Mixed Dentition Stage

**DOI:** 10.1155/2014/109301

**Published:** 2014-10-13

**Authors:** Suresh Ramamurthy, Ramaswamy Satish, Kalidass Priya

**Affiliations:** Department of Orthodontics, Adhiparasakthi Dental College and Hospital, Melmaruvathur, Tamilnadu 603319, India

## Abstract

Fusion is one of the developmental dental anomalies in which two adjacent teeth are joined at the crown level forming a single tooth with an enlarged crown. Fusion causes some clinical problems such as unaesthetic appearance, pain, caries, and malocclusion. The management of fusion often needs multidisciplinary approach to give best possible esthetic and functional outcome. This paper reports a case of 9-year-old boy with fused maxillary left central and lateral incisors who was treated with 2 × 4 fixed orthodontic appliances after surgical separation of fused teeth.

## 1. Introduction 

Tooth fusion is defined as union between two or more separate developing teeth [1]. The union may be between enamel or enamel and dentin. The terms such as synodontia, connate teeth, joined teeth, or double formations are often used to describe fused teeth [1]. Fusion most commonly occurs in the anterior region of primary dentition. It may be seen in unilateral or bilateral region [2]. Usually fusion occurs between two normal teeth and sometimes it is seen between normal tooth and supernumerary tooth.

Fusion can be classified as complete and incomplete type based upon the stage of tooth development. Complete fusion takes place, if the contact occurs before the calcification stage, whereas incomplete takes place at the root level after the formation of crown. The prevalence of fusion in the primary, permanent, and supernumerary teeth is 0.5%, 0.1%, and 0.1%, respectively [3].

The etiology of fusion is still an enigma and many different views have been put forward. Shafer et al. [4] reported that fusion resulting from pressure produced by some physical force prolongs the contact of the developing teeth. Lowell and Soloman believe that fused teeth result from some physical action that causes the young tooth germs to come into contact, thus producing necrosis of the intervening tissue, thus allowing the enamel organ and dental papilla to fuse together [5]. Many authors have also suggested hereditary involvement as an autosomal dominant trait with reduced penetrance [6].

This developmental anomaly may cause clinical problems including esthetic impairment, pain, caries, and tooth crowding [7, 8]. Treatment of fused teeth usually requires multidisciplinary approaches. This case report presents the surgical and orthodontic management of unilaterally fused maxillary left central and lateral incisors in the early mixed dentition stage.

## 2. Case Report

**Figure 1:**
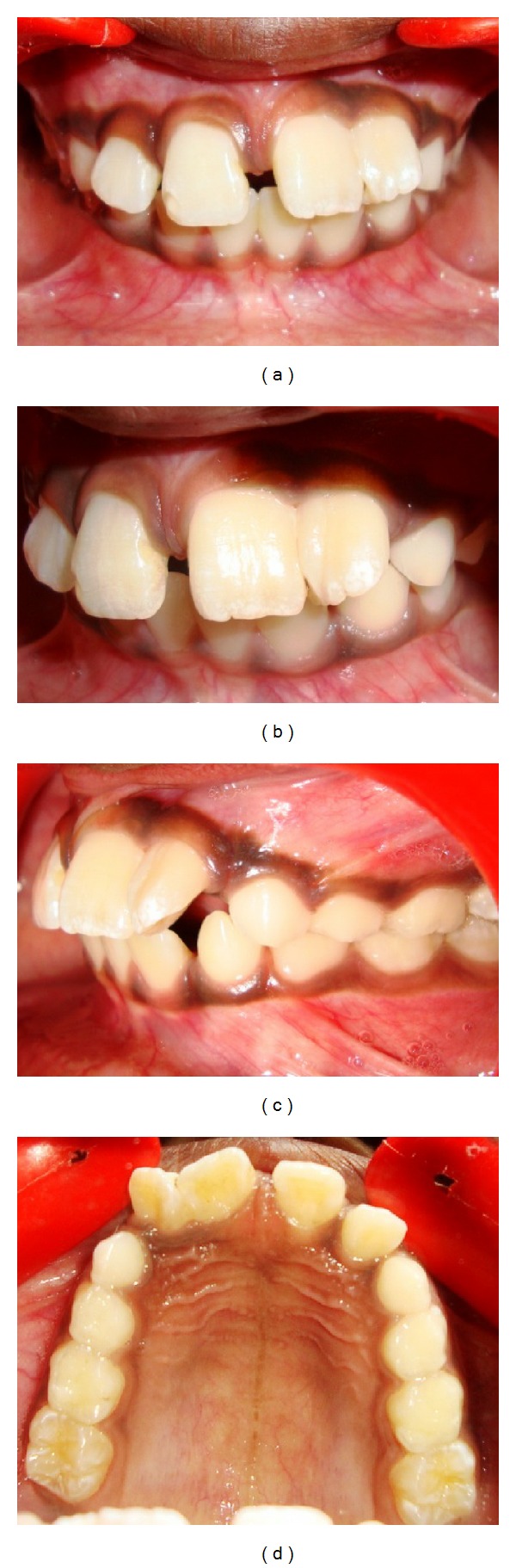
Pretreatment intraoral photos of fused maxillary left central and lateral incisors. (a) Frontal view. (b) Close-up view. (c) Left lateral view. (d) Maxillary occlusal view.

A 9-year-old boy referred to our clinic with complaint of unaesthetic appearance of his upper anterior teeth. The patient had a nonsignificant medical history and no case of fusion was reported in his family. Intraoral examination revealed localized macrodontia present in the maxillary anterior region. Clinically maxillary left central and lateral incisors were found to be fused and indentation running from incisal edge to cervical margin was also observed. Maxillary left central and right lateral incisor crowns were distally tipped. Distolabial rotation of left lateral incisors was present. The labial and palatal aspects of both 21 and 22 teeth were found to be caries free and healthy periodontium. Spacing was present in the maxillary anterior region and distal to lateral incisor in the lower arch (Figure 1).

**Figure 2:**
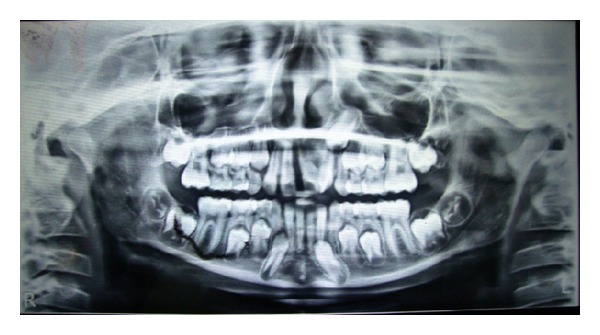
Pretreatment panoramic radiograph showing incomplete fusion of 21 and 22 at crown level.

Orthopantogram radiograph revealed incomplete fusion of 21 and 22 at crown level with separate pulp chambers and two distinct roots. Radiographic evaluation also revealed an interference in eruption of permanent canine 23 by distally tipped root of fused lateral incisor (22) (Figure 2). Treatment was recommended in order to improve esthetic status of the patient, guide the canine into normal eruption path, and intercept developing malocclusion, which may require comprehensive orthodontic treatment in future.

The treatment plan was explained to his family and with their consent; the periodontal envelope flap was raised after anaesthetizing right side of the maxillary anterior region. Initially the fused teeth were separated slightly beyond cementoenamel junction using long, thin diamond bur. After that, an elevator was used to separate the fused teeth; successful separation of 21 and 22 was confirmed by clinical mobility of individual tooth and assessed through radiograph. The periodontal flap was then replaced and suture placed.

**Figure 3:**
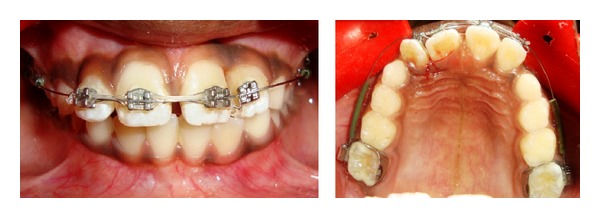
Intraoral view of 2 × 4 fixed appliance.

**Figure 4:**
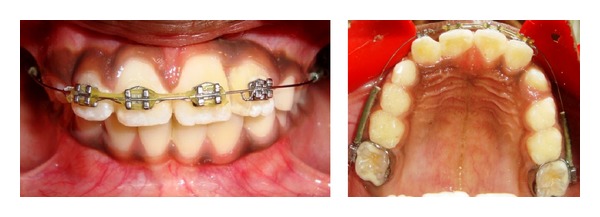
Midtreatment photograph shows correction of fused teeth.

**Figure 5:**
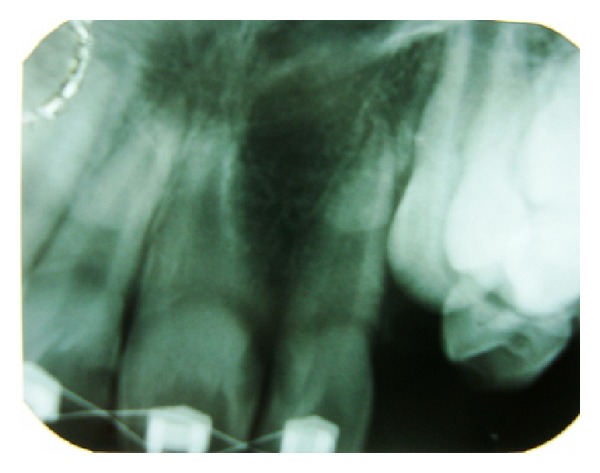
Intraoral periapical radiograph shows root parallelism of 21 and 22.

After a week period, suture was removed and orthodontic treatment with 2 × 4 fixed appliance was begun in maxillary arch. Roth 0.022 slot brackets were bonded on maxillary incisors and preformed molar bands with buccal tube were cemented in 16 and 26. Initially, 0.014 nickel titanium archwire with protective sleeve was used for alignment and leveling (Figure 3), after that progressively archwires were changed to 0.016 nickel titanium and 0.018 stainless steel wire. Maxillary anterior spaces were closed with elastomeric chain in 0.018 stainless steel wire. Fused teeth were aligned at the end of six-month orthodontic treatment (Figure 4). Intraoral periapical radiograph showed improvement in root parallelism of 21 and 22 during fixed appliance treatment (Figure 5).

## 3. Discussion

Developmental anomalies of teeth may occur due to abnormalities in the differentiation of the dental lamina and the tooth germs or abnormalities in the formation of the dental hard tissues [9]. Different terminologies have been used to describe the anomaly of double teeth such as fusion, gemination, and twinning [10]. Isolated large teeth may be the result of union of two adjacent tooth buds or partial splitting of one into two. Gemination is defined as an attempt of single tooth bud to divide with the resultant formation of a tooth with a bifid crown and usually a common root and root canal. Fusion is the union of two normal teeth with separate tooth buds leading to the formation of a joined tooth with confluence of dentin. The term twinning has sometimes been used to designate the production of identical structures by division resulting in one normal and one supernumerary tooth. Various authors prefer to use the term twinning or double tooth to describe fusion and gemination because of difficulty in differentiating the two conditions [11].

Still confusion presents in differentially diagnosing fusion and gemination clinically which are two different morphological dental anomalies, characterized by the formation of a large size tooth. Careful radiographical and clinical examinations are required to separate these two anomalies [12]. Madder's two tooth rule may be a practical way of differentiating fusion and gemination. If fused tooth are counted as one and the number of teeth in the dental arch is less, then the term fusion is considered. However, when the abnormal tooth is counted as one and the number of teeth in dental arch is normal, then it is termed as gemination or is a case of fusion between normal and supernumerary teeth. This case revealed fusion of two teeth involving the coronal surfaces with two separate roots and distinct pulp chambers and canals.

The clinical problems associated with fusion include abnormal shape of the tooth leading to unaesthetic appearance, delayed exfoliation, occlusal disturbances, and space discrepancies. The presence of fissures or grooves at the union between fused teeth predisposes to caries and periodontal disease.

The treatment of fusion depends upon the patients esthetic and functional requirements. Three ways of treating incomplete fusion are as follows: crown width of the fused teeth reduced by selective grinding, surgical sectioning followed by restoration of teeth structures to normal size, and separation and extraction of the anomalous tooth with orthodontic closing of the space and reshaping of the teeth [13, 14]. In this case, fused teeth were successfully separated with long thin bur and esthetic was improved with orthodontic treatment.

## 4. Conclusion

Early identification and timely management of fused teeth may prevent orthodontic, periodontal, and endodontic complications. Treating fusion in young patient is easy and helps to improve self-esteem and quality of life in future.
